# Characterisation of fibrosis in chemically-induced rat mammary carcinomas using multi-modal endogenous contrast MRI on a 1.5T clinical platform

**DOI:** 10.1007/s00330-017-5083-6

**Published:** 2017-10-16

**Authors:** Neil P. Jerome, Jessica K. R. Boult, Matthew R. Orton, James A. d’Arcy, Ashutosh Nerurkar, Martin O. Leach, Dow-Mu Koh, David J. Collins, Simon P. Robinson

**Affiliations:** 1 CR-UK Cancer Imaging Centre, Division of Radiotherapy and Imaging, The Institute of Cancer Research, London, SM2 5NG UK; 2 Department of Histopathology, Royal Marsden NHS Foundation Trust, London, SW3 6JJ UK; 3 Department of Radiology, Royal Marsden NHS Foundation Trust, London, SM2 5PT UK

**Keywords:** Functional magnetic resonance imaging, Magnetisation transfer contrast imaging, Fibrosis, Mammary neoplasms, Mammary carcinoma, animal

## Abstract

**Objectives:**

To determine the ability of multi-parametric, endogenous contrast MRI to detect and quantify fibrosis in a chemically-induced rat model of mammary carcinoma.

**Methods:**

Female Sprague-Dawley rats (n=18) were administered with *N*-methyl-*N*-nitrosourea; resulting mammary carcinomas underwent nine-b-value diffusion-weighted (DWI), ultrashort-echo (UTE) and magnetisation transfer (MT) magnetic resonance imaging (MRI) on a clinical 1.5T platform, and associated quantitative MR parameters were calculated. Excised tumours were histologically assessed for degree of necrosis, collagen, hypoxia and microvessel density. Significance level adjusted for multiple comparisons was p=0.0125.

**Results:**

Significant correlations were found between MT parameters and degree of picrosirius red staining (r > 0.85, p < 0.0002 for k_a_ and δ, r < -0.75, p < 0.001 for T_1_ and T_1s_, Pearson), indicating that MT is sensitive to collagen content in mammary carcinoma. Picrosirius red also correlated with the DWI parameter fD* (r=0.801, p=0.0004) and conventional gradient-echo T_2_* (r=-0.660, p=0.0055). Percentage necrosis correlated moderately with ultrashort/conventional-echo signal ratio (r=0.620, p=0.0105). Pimonidazole adduct (hypoxia) and CD31 (microvessel density) staining did not correlate with any MR parameter assessed.

**Conclusions:**

Magnetisation transfer MRI successfully detects collagen content in mammary carcinoma, supporting inclusion of MT imaging to identify fibrosis, a prognostic marker, in clinical breast MRI examinations.

**Key Points:**

*• Magnetisation transfer imaging is sensitive to collagen content in mammary carcinoma.*

*• Magnetisation transfer imaging to detect fibrosis in mammary carcinoma fibrosis is feasible.*

*• IVIM diffusion does not correlate with microvessel density in preclinical mammary carcinoma.*

**Electronic supplementary material:**

The online version of this article (10.1007/s00330-017-5083-6) contains supplementary material, which is available to authorized users.

## Introduction

Breast cancer development and growth is strongly influenced by the crosstalk of tumour cells with the surrounding extracellular matrix/stroma [1–3]. The stroma can make up a significant proportion of a breast carcinoma [4], and differs from normal stroma, bearing closer resemblance to granulation tissue and wound healing, with a high number of fibroblasts, deposition of type I collagen and fibrin, and the infiltration of inflammatory cells [5]. The presence of a fibrotic focus, a central scar-like area within a carcinoma that represents a focus of exaggerated reactive tumour stromal formation, was first proposed as an indicator of increased tumour aggressiveness in invasive ductal breast cancer by Hasabe et al. [6], and has since been linked to early disease relapse, lymph node and osteolytic bone metastasis, and reduced long-term survival [7–9]. Hypoxia has also been associated with the formation of fibrotic foci [5].

Advanced MRI techniques provide a means of defining non-invasive quantitative biomarkers to inform on biologically relevant structure-function relationships in tumours, thereby enabling an understanding of their behaviour and heterogeneous distribution [10]. Imaging biomarkers for assessing tumour pathophysiology require evaluation before being routinely deployed in clinical trials; in particular, imaging-pathology correlation, and thus whether the imaging biomarker reflects underlying pathology is important to establish, but can often only meaningfully be studied in animal models [11].

The newly generated tumour stroma shows similarities with granulation tissue and subsequent scar formation in wound healing and differs from the normal stroma by an increased number of fibroblasts, enhanced capillary density, deposition of type I collagen and fibrin and the presence of inflammatory cells.

Several MRI biomarkers have the potential to detect breast cancer fibrosis. The fibrous nature of collagen may increase the non-monoexponential contribution to the diffusion-weighted MRI (DWI) signal, arising from the propensity of water molecules to diffuse along the fibres, combined with reduced diffusivity from encountering more barriers to random diffusion, compared to surrounding tissue [12–14]. Increased macromolecular collagen fibre content may also yield a greater destruction of signal arising from MT MRI from off-resonance saturation [15], and the short-lived signal of collagen (T_2_*~500μs) may be detectable with ultrashort-echo time (UTE) sequences [16]. Dynamic contrast-enhanced (DCE) MRI remains a standard technique used in breast cancer MRI protocols and may be suitable for fibrosis detection in some tissues [17], but the use of contrast adds complexity to clinical studies and can be contraindicated in certain patients.

This study aims to determine the ability of multi-parametric MRI incorporating several endogenous contrast mechanisms, such as DWI, MT-MRI and UTE-MRI, performed on a clinical imaging platform, to detect and quantify fibrosis in a chemically-induced rat model of mammary carcinoma previously shown to produce heterogeneous tumours with a range of fibrosis severity [18].

## Materials and methods

### Animal procedures

This study was performed in accordance with the local ethics review panel, United Kingdom National Cancer Research Institute guidelines for animal welfare in cancer research, and the ARRIVE (Animal Research: Reporting In Vivo Experiments) guidelines [19, 20]. Female Sprague-Dawley rats (200–250 g, n=18; Charles River, Margate, UK) were injected with 37.5 mg.kg^−1^ of refrigerated *N*-methyl-*N*-nitrosourea (MNU, Sigma-Aldrich, Poole, UK) intraperitoneally, resulting in tumours that spontaneously developed at various sites associated within the mammary fat-pad [18]. Tumour formation was detected by palpation and growth was monitored by calliper measurement; animals were imaged when tumours reached approximately 3cm^3^ (using ellipsoid volume formula, (π/6)×L×W×D).

Animals were anaesthetised using a 4 ml.kg^-1^ intraperitoneal injection of fentanyl citrate (0.315 mg.ml^-1^) plus fluanisone (10 mg.ml^-1^ (Hypnorm; Janssen Pharmaceutical Ltd., High Wycombe, UK)), midazolam (5 mg.ml^-1^ (Hypnovel; Roche)), and water (1:1:2). Prior to imaging, an intraperitoneal injection of 60 mg.kg^-1^ pimonidazole (Hypoxyprobe, Burlington, VT, USA) in phosphate- buffered saline was given, in preparation for subsequent histological staining for hypoxia.

### Magnetic resonance imaging

MR imaging was performed on a MAGNETOM Avanto 1.5T clinical scanner (Siemens Healthcare, Erlangen, Germany), to validate clinical sequences and support methodological transfer. For MRI, the animal was secured supine using an insulating vacuum beanbag to both retain body heat and prevent excessive movement. The animal was placed with the tumour centred on top of a small-loop temporomandibular joint (TMJ) coil, itself centred within the multi-element head receiver coil [21]. Elements of the head coil array were used in parallel with the small-loop coil during all acquisitions. Scans were performed in the coronal plane, with full tumour coverage. Morphological T_2_-weighted fast spin-echo images were obtained for anatomical localisation. Diffusion-weighted MRI (DWI), ultrashort-echo time (UTE) MRI and magnetisation transfer (MT) data were acquired centred on the lesion.

UTE data were acquired with a prototype three-dimensional (3D) multiple gradient echo (mGRE) sequence with 1.1 mm isotropic resolution; the first echo acquired was on the free induction decay (FID) immediately following the read pulse, followed by four regular gradient echoes. This acquisition was repeated in order to acquire four ultrashort-echo times (70–560 μs). DWI was based on a clinical patient protocol (nine b-values, 0–800 mm^-2^s; see Table 1) acquired in free-breathing using a fat-suppressed two-dimensional (2D) single-shot prototype EPI sequence. MT data were acquired as a series of matched 3D GRE acquisitions, with 1.0-mm isotropic voxels, and two flip angles with/without an MT pulse set at 1.5 kHz offset. Detailed sequence parameters are given in Table 1, and were adapted from clinical imaging sequences; the total acquisition time was approximately 1 hour.

**Table 1:** MR imaging parameters for anatomical imaging (T_2_w), diffusion-weighted imaging (DWI), ultrashort-echo time imaging (UTE) and magnetisation transfer imaging (MT). Total protocol time: approx. 1 h

Modality	T_2_w	DWI	UTE	MT
Sequence type	TSE	2D EPI	3D mGRE	3D GRE
Slices	24	18	96	30
FOV (mm)	120×72	150×105	103×103	128×96
Slice thickness (mm)	1	1.5	1.07	1
Matrix Size	256×152	102×72	96×96	128×96
TR (ms)	800	2100	42	15
TE(ms)	9.6	60.8	7.16, 11.64, 16.12, 20.60	2.52
UTE (ms)	-	-	0.07, 0.14, 0.28, 0.56	-
NSA	1	18	1	8
iPAT	GRAPPA 2	GRAPPA 2	-	GRAPPA 2
Fat sat.	No	Yes	Yes	No
b-values (mm^-2^s)	-	0, 20, 40, 60, 80, 100, 200, 400, 800	-	-
Variable flip angles (°)	-	-	-	4°, 24°
MT pulse	-	-	-	without/with (1.5 kHz)
Time (min:s)	4:54	15:58	4 × 5:36	4 × 2:28

### MR image analysis

MRI analysis was performed using proprietary software (ADEPT, The Institute of Cancer Research, London, UK). All MR images were reviewed, and regions of interest (ROIs) were independently drawn by two observers, MR scientists (NPJ and DJC) with 5 and 32 years’ experience, respectively, in conducting preclinical MR studies. Repeatability of ROI delineation was assessed using the Sørenson-Dice similarity coefficient. Each ROI was drawn around the tumour on the imaging slice that macroscopically matched the histological section stained, MR parameters calculated on a voxel-by-voxel basis, and reported as the average value for repeated ROI median values per slice analysed together with calculation of repeat-measures coefficient of variation (CoV).

For DWI analysis, the perfusion-insensitive apparent diffusion coefficient (ADC) was estimated using images for b=200 mm^-2^s and above [22], with a single-exponential model (Eq. 1). All b-values were used for intravoxel incoherent motion (IVIM) fitting using a bi-exponential model (Eq. 2) to simultaneously derive estimates of pseudodiffusion fraction (f), pseudodiffusion coefficient (D*) and tissue diffusivity (D). The compound parameter fD* was also calculated. Initial estimate values for IVIM fitting were found using the segmented approach [22], by estimating D using a monoexponential fit of images with b=200 mm^-2^s and above (as per ADC) and f from the observed S_0_ relative to the intercept of this curve at b=0 mm^-2^s:

1 $$ {S}_b-{S}_o.\exp \left(-b. ADC\right) $$

2 $$ {S}_b={S}_o.\left[f.\exp \left(-b.{D}^{\ast}\right)+\left(1-f\right).\exp \left(-b.D\right)\right] $$

where the observed signal intensity at a given b-value is denoted S_b_, and S_0_ is the corresponding signal at b=0mm^-2^s (equal to the total available signal S_total_ modulated by the apparent T_2_ and the acquisition echo time, S_0_=S_total_.exp(-TE/T_2app_) [23].

For UTE imaging, T_2_*_short_ was calculated using the first (ultrashort, < 1ms; see Table 1 for values) echo from successive imaging acquisitions, and the conventional T_2_*_long_ using the remaining (i.e. not ultrashort) echoes from all acquisitions, using separate mono-exponential models (Eq. 3); the ratio of the calculated signal arising, analogous to f in the IVIM DWI model, from each of the two relaxation constants was also calculated. All DWI and UTE fitting was performed using a Markov Chain Monte Carlo (MCMC) Bayesian statistical approach [24] as a robust least-squares estimator, with no data filtering.

3 $$ {S}_{TE}={S}_o.\exp \left(-\frac{TE}{T_2^{\ast }}\right) $$

MT acquisition images were used for calculation of magnetisation transfer ratio (MTR) (Eq. 4) [25, 26], longitudinal relaxation constants in the presence/absence of the MT pulse using the variable flip angle (VFA) method [27] (T_1_ and T_1s_, respectively), and B_1_-independent MT saturation (δ) and apparent MT rate (k_a_) (Eqs. 5 and 6) [26, 28]:

4 $$ MTR=\left({S}_{ref}-{S}_{MT}\right)/{S}_{ref} $$

5 $$ {k}_a= MTR/{T}_{1s} $$

6 $$ \delta =\left({R}_{1 app} TR+{\alpha_{nom}}^2/2\right)\left({S}_{ref}-{S}_{MT}\right)/{S}_{MT} $$

where S_ref_ and S_MT_ are signal amplitudes from identical sequences acquired with and without the MT pulse, TR is the acquisition repetition time, R_1app_ is the spin-lattice relaxation rate (or T_1_^-1^), α_nom_ is the nominal acquisition flip angle in radians, and the small flip angle approximation is used [26].

### Histological staining and analysis

Following MR imaging, animals were killed by cervical dislocation, and the tumour excised and fixed in 10 % formalin. Tumours were then cut through the centre, and embedded in paraffin blocks, with orientation matched to the geometry of the imaging slices to facilitate subsequent image correlation.

Tumour sections (5 μm) were stained with haematoxylin and eosin (H&E), to allow assessment of necrosis and tumour grade, and picrosirius red, to assess collagen I/III deposition (fibrosis). Immunohistochemistry visualised using DAB was performed using FITC-conjugated mouse monoclonal antibodies against pimonidazole adducts, followed by rabbit anti-FITC antibodies, for the detection of hypoxic regions, or rabbit monoclonal antibodies against CD31 (EP3095; Millipore, Watford, UK) to assess vascular endothelial cells as a proxy for perfusion. Whole tumour images were acquired using a motorised scanning stage (Prior Scientific Instruments, Cambridge, UK) attached to a BX51 microscope (Olympus Medical, Southend-on-Sea, UK) driven by CellP (Soft Imaging System, Munster, Germany). Snapshots at ×200 magnification were also acquired from CD31-stained sections.

Tumour grade and degree of necrosis (semi-quantitative assessment) were evaluated by an expert pathologist (AN). Percentage area of each tumour section displaying pimonidazole adduct or picrosirius red positivity was measured using pixel counts from a customised routine operating on a Lab colour-space separation into stain and non-stain classes (Mathworks, Natick, MA, USA) of a digital image, and visually confirmed for accuracy. Microvessel density was assessed by counting CD31-positive vessels from six random fields (×200) distributed across the section and the number converted to vessels/mm^2^.

## Statistics

MRI-derived parameters are given as the median of the ROI voxels in each observation/analysis, in order to minimise the contribution of outliers arising from partial volume effects. Correlations between MRI markers and histological analyses acquired from matched slices were assessed using Pearson correlation coefficients (r). Bonferroni correction for multiple comparisons against the different histological markers was applied, with results considered significant at p < 0.0125. A partial least-squares regression (PLSR) approach was applied to derived MR parameters for the response variable of picrosirius red stain, to assess the relative performance of a multiparametric approach. Leave-one-out cross-validation (LOOCV) was used to derive normalised root-mean-square error (NRMSE) as a proxy for goodness of response variable prediction.

## Results

### Tumour cohort

Tumours developed in a heterogeneous manner in the mammary fat pad of 15 rats, with imaging performed at an average tumour volume of 3.6±2.1 cm^3^ (average ROI slice area for analysis 338±168 mm^2^) over a wide timeframe post-injection of MNU (median 421 days, range 105–471). One animal simultaneously developed two tumours; both were imaged and analysed. Histology from two tumours was not satisfactorily matched to the imaging plane and was excluded from the analysis. A small sub-cohort (n=4) of the largest tumours was sectioned in two places, into equally sized sections (> 5 mm thick), making 18 matched MR and histological data sets for analysis, from 14 tumours in 13 rats (one animal with two tumours, and two distinct regions each from four tumours).

### Histological slice matching and analysis

**Fig. 1:**
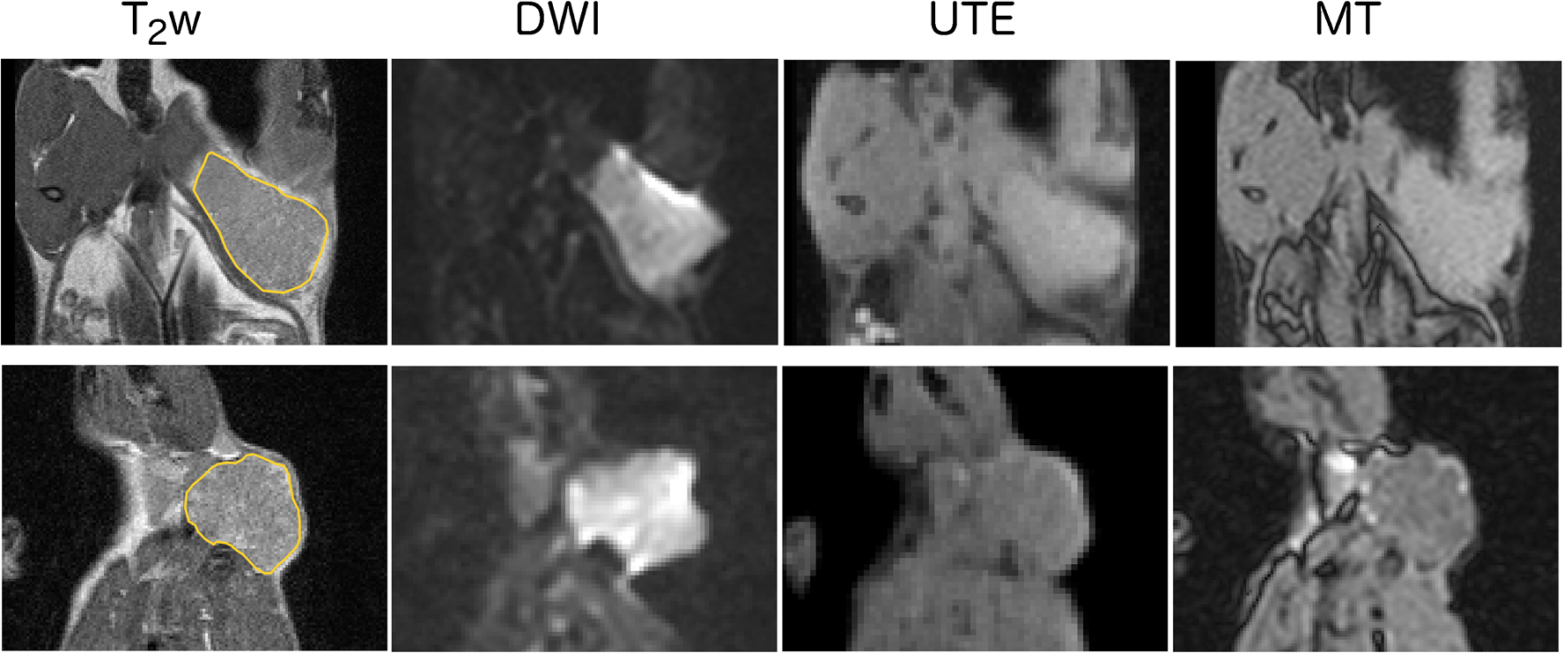
Representative anatomical and functional images from two *N*-methyl-*N*-nitrosourea-induced rat mammary carcinomas, showing the variation in tumour presentation and typical images from the multiparametric MRI strategy used herein: (a) T_2_-weighted morphological imaging (T_2_w), (b) diffusion-weighted imaging (DWI; b=0 mm^-2^s), (c) ultrashort-echo time imaging (UTE; TE=0.07ms), and (d) magnetisation transfer imaging (MT; flip angle 4°, with MT pulse)

**Fig. 2:**
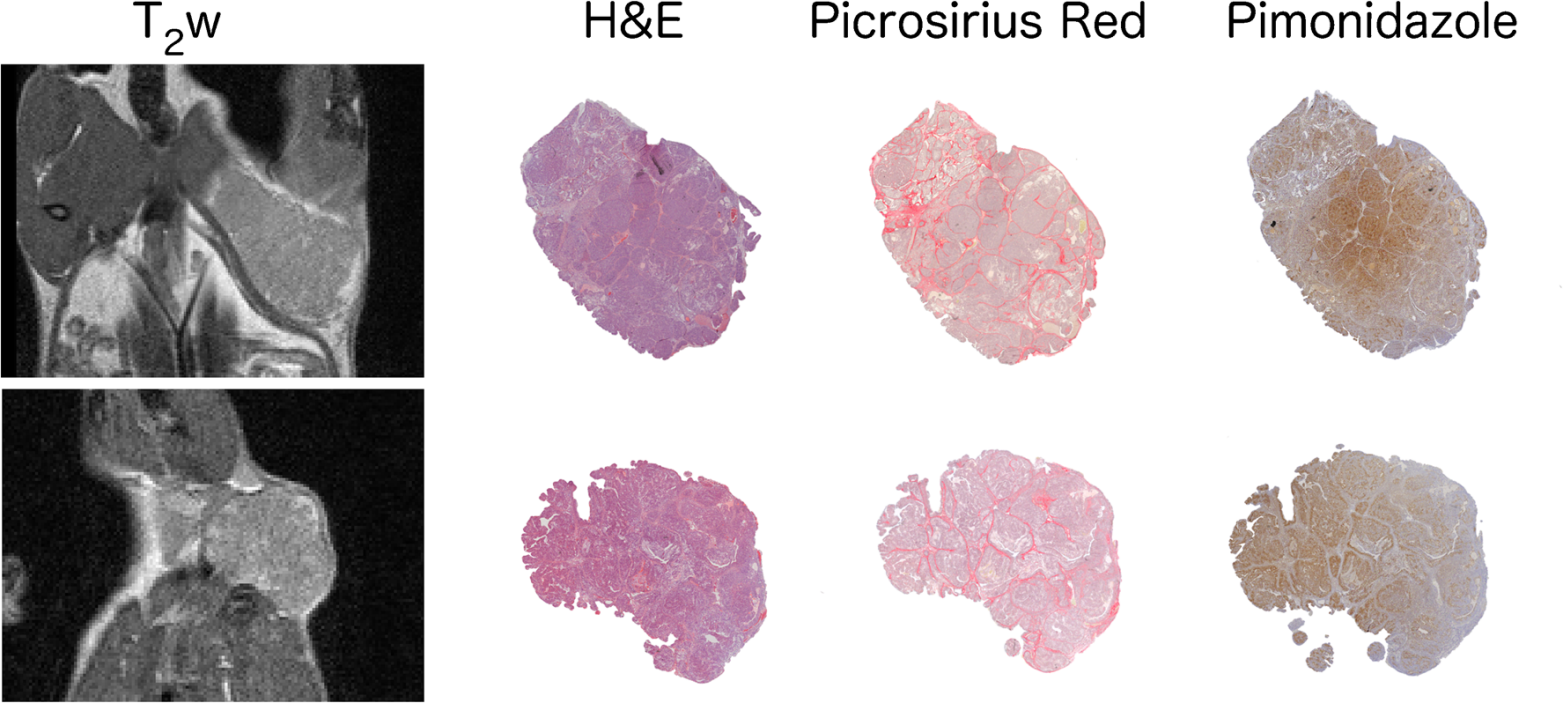
Representative images from the same tumours as shown in Fig. 1 (upper row tumour 1, section 1, and lower row tumour 3 section 2; see Table 2 for analysis) showing matching of MRI with histology (left to right) T_2_-weighted MRI, H&E staining, picrosirius red staining for collagen I/III and pimonidazole adduct immunohistochemistry for hypoxia

Representative anatomical and functional images from two tumours are shown in Fig. 1, highlighting the varying contrast and resolution (including a typical ROI for analysis) obtained for each biomarker using the multiparametric MRI approach. The use of different slice thicknesses meant that the MR slice locations were not identical, but in each case were the closest match for the associated histology. Visual matching of the MRI with the corresponding histological sections was good, as demonstrated in Fig. 2 (same tumours as shown in Fig. 1).

Colour segmentation of picrosirius red staining and pimonidazole adduct immunohistochemistry successfully and robustly separated the desired stain from the remaining tissue and background (Supplementary Fig. S1), with repeated segmentation for picrosirius red staining giving essentially identical results (correlation 0.98, p > 0.0001). Details of the tumour cohort, including time from MNU injection to imaging (days) and tumour volume alongside histologically assessed tumour grade, degree of necrosis, microvessel density (MVD), percentage pimonidazole adduct formation and percentage picrosirius red staining, are given in Table 2.

**Table 2:** Cohort characteristics for animals with *N*-methyl-*N*-nitrosourea-induced mammary carcinoma

Animal	Section	Days at scan	Volume (cm^3^)	Tumour grade	Necrosis (%)	Microvessel density (vessels/mm^2^)	Pimonidazole (%)	Picrosirius red (%)
1	1	105	5.3	1	5	115	27.7	23.1
	2			1	5	104	28.5	9.8
2	1	126	7.3	1	15	128	17.2	12.0
	2			1	10	121	14.9	12.1
3	1	129	4.5	2	5	60	28.0	7.8
	2			2	5	150	21.2	9.8
4		189	0.9	1	20	38	23.2	10.4
6		332	4.8	1	1-2	65	23.2	52.3
7		372	2.6	3	5	114	19.9	16.8
8	1	421	3.6	1	0	68	32.1	28.1
	2			1	1-2	120	33.6	32.2
10		436	2.3	2	5	176	47.4	7.2
11		436	1.8	2	15	62	44.1	35.9
12		449	5.6	2	1-2	100	21.6	32.8
13-1		457	2.9	2	0	45	27.9	19.3
13-2			2.8	2	0	69	27.7	50.0
14		470	0.6	2	0	45	14.5	80.4
15		471	0.3	3	5	116	12.8	27.1

### Repeatability of ROIs and MR parameters

The Sørenson-Dice similarity coefficients of the ROIs drawn by the two observers ranged between 0.72 and 0.96, with a median of 0.89 across all ROIs and no less than 0.88 within each MR modality, which demonstrates excellent agreement between observers. Repeat-measures percentage coefficients of variation (CoV) for positive-constrained MR-derived parameters were calculated using log-transformed values [29]. Excellent repeatability was shown for all MR parameters, with CoV values ranging from 1.5 % to 8.6 % (see Table 3), with the notable exception of IVIM f and D*, which are known to display poor repeatability [30].

**Table 3:** Correlation coefficients, r, between MR and histological parameters of matched slices

MR modality	MR parameter (repeatability CoV, %)	Histological parameter (*p*-value in parenthesis; **bold** indicates significance, *p* < 0.0125)			
		Necrosis	MVD	Pimonidazole adduct	Picrosirius red
DWI	ADC (6.14)	0.328 (0.232)	0.185 (0.5104)	-0.078 (0.7833)	-0.568 (0.0274)
	D (5.18)	0.309 (0.2627)	0.207 (0.4587)	-0.099 (0.7244)	-0.574 (0.0253)
	f (39.82)	-0.607 (0.0165)	-0.269 (0.3325)	0.134 (0.6349)	0.556 (0.0314)
	D* (58.42)	0.556 (0.0313)	-0.070 (0.8045)	-0.172 (0.5407)	-0.035 (0.9026)
	fD* (22.24)	-0.552 (0.033)	-0.449 (0.0933)	0.071 (0.8021)	**0.801 (0.0004)**
UTE	T_2_*_long_ (5.69)	0.240 (0.3701)	0.409 (0.1303)	0.203 (0.4501)	**-0.660 (0.0055)**
	T_2_*_short_ (8.58)	-0.274 (0.3038)	0.305 (0.269)	0.085 (0.7537)	-0.092 (0.734)
	ratio (1.77)	**0.62 (0.0105)**	0.291 (0.2927)	0.155 (0.567)	-0.290 (0.2764)
MT	MTR (1.57)	-0.219 (0.4142)	-0.362 (0.1851)	0.06 (0.8241)	0.575 (0.0198)
	T_1_ (1.49)	0.418 (0.1075)	0.377 (0.1661)	0.455 (0.0763)	**-0.758 (0.0007)**
	T_1s_ (7.75)	0.363 (0.1673)	0.363 (0.1832)	0.340 (0.1981)	**-0.831 (0.0001)**
	k_a_ (7.88)	-0.367 (0.1623)	-0.357 (0.1912)	-0.234 (0.3831)	**0.857 (0.0001)**
	δ (6.70)	-0.521 (0.0387)	-0.537 (0.0391)	-0.209 (0.4364)	**0.869 (0.0001)**

*ADC* apparent diffusion coefficient, *MVD* microvessel density, *DWI* diffusion-weighted imaging, *UTE* ultrashort-echo time imaging, *MT* magnetisation transfer imaging

### Diffusion-weighted imaging

**Fig. 3:**
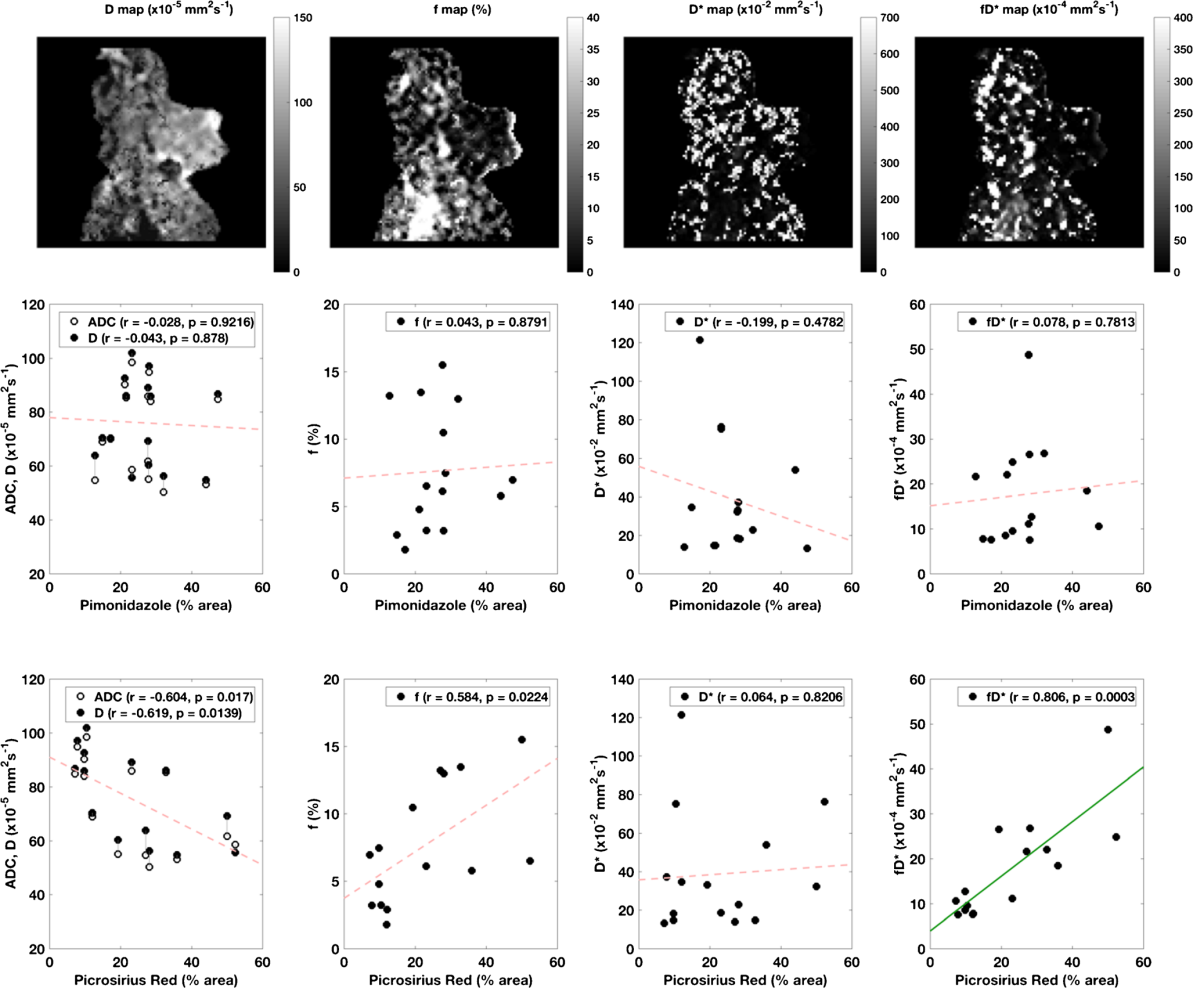
Example maps (top row) for the fitted intravoxel incoherent motion (IVIM) functional parameters, with a binary mask to exclude pure noise, alongside scatter graphs of diffusion-weighted imaging parameters determined using the apparent diffusion coefficient (ADC) and IVIM models plotted against percentage pimonidazole adduct formation (middle row) and picrosirius red (bottom row) staining. Correlation coefficients and *p*-values are given with each plot, with significant correlations (defined as p < 0.0125, corrected for multiple comparisons) found between IVIM fD* and picrosirius red (unbroken green lines). The combined plot for ADC and D indicates corresponding values; for clarity, only the regression line for D is shown

Scatter graphs of the diffusion parameters derived from both the ADC and IVIM models, plotted against the histological markers, are shown in Fig. 3 alongside example fitted parameter maps. A correlation of r=0.801 (p=0.0004) was found between the pseudodiffusion-related parameter fD* and picrosirius red. Correlation of picrosirius red with the pseudodiffusion fraction f was non-significant under multiple comparison correction (r=0.556, p=0.0314). The IVIM D and monoexponential ADC parameters, notionally reporting on the same true diffusion phenomenon, were highly correlated (r=0.97, p<0.001) as expected; both showed negative correlation with picrosirius red stain (r=-0.574 and -0.568 respectively; significance was not achieved under Bonferroni correction), but not with any other marker. IVIM pseudodiffusion parameters also correlated with percentage necrosis, though none were significant after multiple comparison correction (f: r=-0.607, p=0.0165; D*: r=0.556, p=0.0313; fD*: r=-0.552, p=0.033). The challenge of repeatably fitting pseudodiffusion parameters is reflected in the larger CoV. Correlation coefficients and *p*-values for comparisons of all MR parameters with histological markers are given in Table 3.

### Ultrashort-echo time imaging

**Fig. 4:**
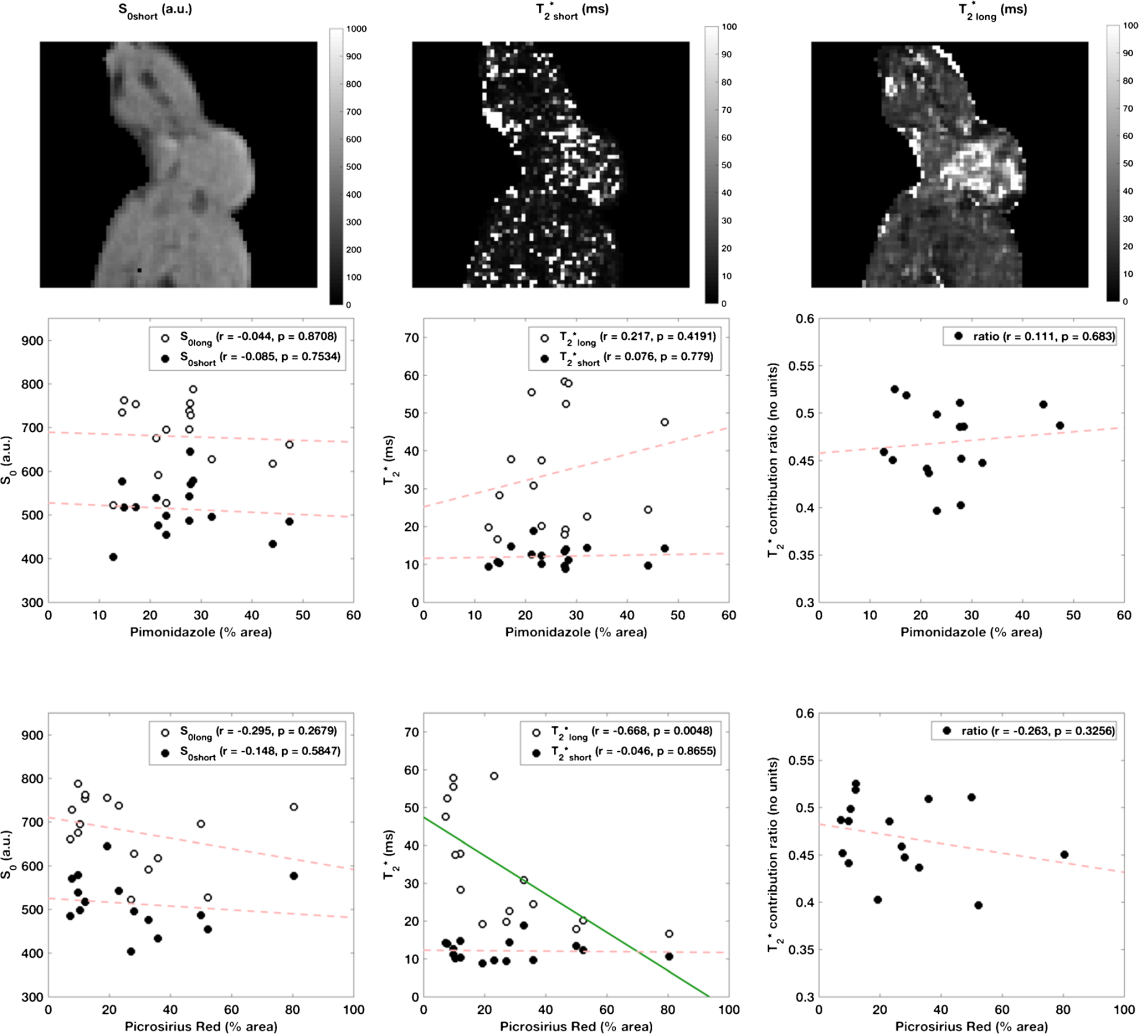
Example maps (top row, with binary mask around animal) from fitting monoexponential model for T_2_* using conventional (T_2_*_long_) and ultrashort-echoes (T_2_*_short_). T_2_* parameters derived from ultrashort-echo time imaging, plotted against percentage pimonidazole adduct (middle row) and picrosirius red (bottom row) staining. Correlation coefficients and *p*-values are inset on each plot (significance defined as p < 0.0125, corrected for multiple comparisons, also indicated by an unbroken green regression line). The T_2_*_long_, calculated from images with TE > 7 ms, shows correlation with picrosirius red staining

Typical parameter maps and data from UTE are shown in Fig. 4. There were no significant correlations with either CD31 or pimonidazole adduct staining; significant correlation was observed only for T_2_*_long_ with picrosirius red (r=-0.660, p=0.0055).

### Magnetisation transfer imaging

**Fig. 5:**
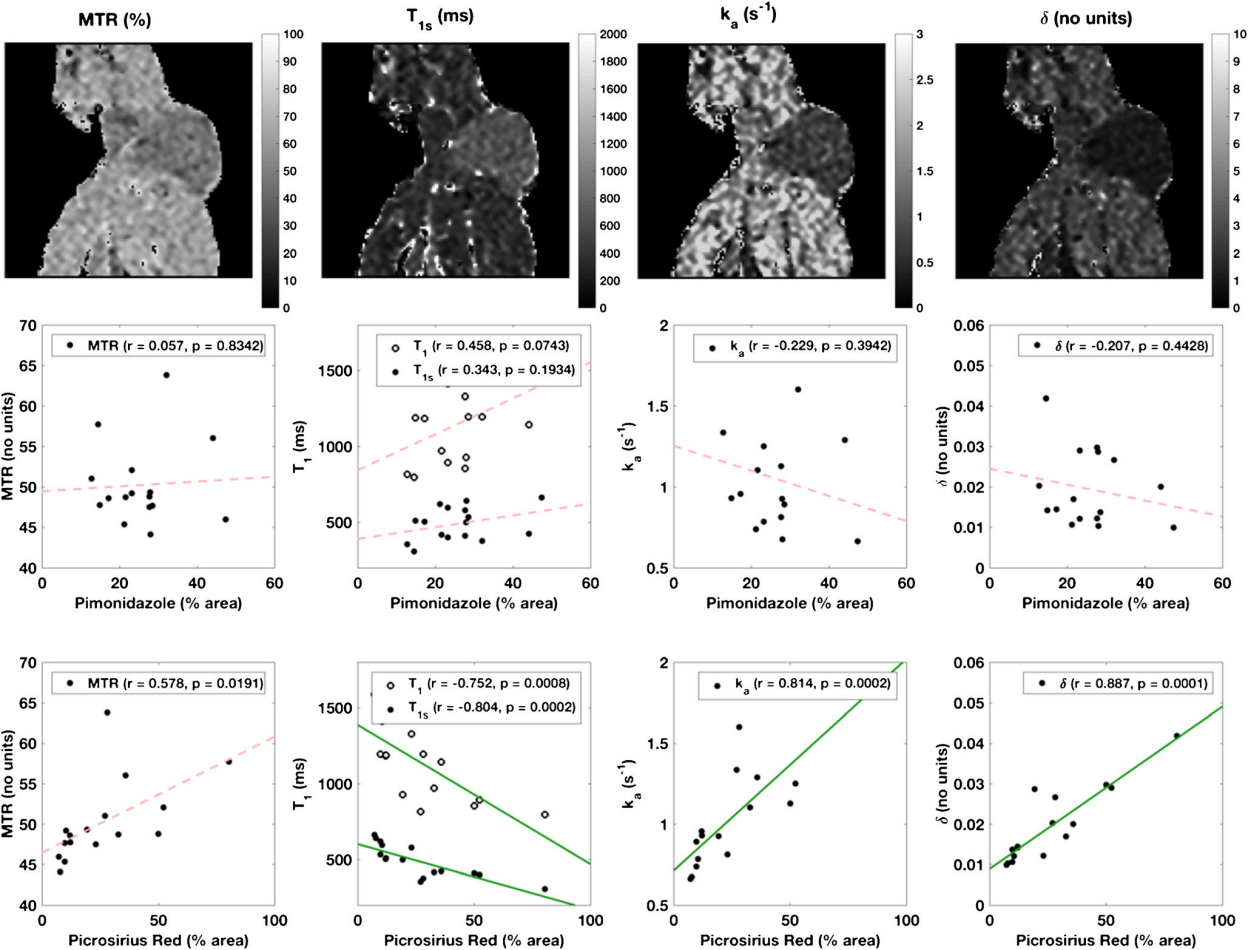
Example magnetisation transfer parameter maps (top row, with binary mask around animal), with corresponding scatter plots with percentage pimonidazole adduct (middle row) and picrosirius red (bottom row) staining, including correlation coefficients and *p*-values (inset). Significant (p < 0.0125, corrected for multiple comparisons) correlations were found for all magnetisation transfer (MT) parameters (unbroken green regression lines) except magnetisation transfer ratio (MTR) with percentage picrosirius red staining

Correlations of the MT parameters with histological markers are shown in Fig. 5, alongside typical parameter maps. There were significant correlations for all MT parameters, excluding the B_1_-dependent measure MTR, with the percentage of picrosirius red staining. T_1_ and T_1s_ showed similar negative correlations (r=-0.758 and -0.831, respectively, p<0.001), with decreased T_1_ correlated to increased picrosirius red stain. MTR had the weakest positive correlation (r=0.575, p=0.0198), whereas accounting for B_1_-dependence in δ gave a stronger correlation (r=0.869, p=0.0001). The apparent MT rate constant k_a_ was also significantly correlated with picrosirius red (r=0.857, p=0.0001). CD31 and estimated necrosis also correlated with δ (r=-0.537, p=0.0391 and r=-0.521, p=0.0387, respectively) but these were weaker and not significant following multiple comparison correction.

### Multiparametric partial least squares regression analysis

The normalised root-mean-squared error (NRMSE) from LOOCV of linear regression for each of the MT parameters are presented in Table 4, and correspond to the observed correlations (Fig. 5). Conducting a PLSR analysis using all five MT parameters yielded a single-variable model, with loadings corresponding to the observed correlations, and a comparable NRMSE indicating that combinations of MT parameters do not necessarily outperform individual correlations. The corresponding PLSR using parameters across all MR modalities similarly gave a single LV model with a NRMSE that did not benefit from inclusion of other MR modalities, although DWI parameters ADC and D contributed to LV1. Loadings plots are shown in Supplementary Fig. S2 for both analyses.

**Table 4:** Comparison of single magnetisation transfer imaging (MT) parameters (1–5) with partial least squares regression analysis of (i) all MT, and (ii) all MR-derived parameters (latent variable details in Supplementary Fig. S2)

Variable	Model parameter	LOOCV NRMSE
1	MTR	0.3231
2	T_1_	0.1930
3	T_1s_	0.1677
4	k_a_	0.1663
5	δ	0.1581
All MT (n=5)	LV1	0.1731
All MR (n=11)	LV1	0.2513

*LOOCV* leave-one-out cross-validation, *NRMSE* normalised root mean square error

## Discussion

The presence of a histologically-confirmed fibrotic focus has been shown to be a predictor of increased tumour aggressiveness, relapse, metastasis and poor long-term survival in breast cancer [6–9]. Fibrotic foci are also associated with tumour hypoxia, an independent indicator of poor treatment response and prognosis [5, 18, 31–33]. The ability to detect fibrosis within mammary carcinomas non-invasively would be of great value in helping guide personalised treatment. The validation of appropriate MRI techniques with potential to inform on fibrosis using preclinical models with matched histology can directly guide development of imaging studies in the clinical setting.

In this study, a range of endogenous MR imaging contrasts were measured in chemically-induced mammary carcinomas arising in rats injected with MNU; the tumours were highly heterogeneous and presented with a range of fibrosis levels as previously observed in this model and typical of the clinical setting [18, 34]. The imaging performed in the study used exclusively clinical hardware, conferring greater translational relevance to the study, and the scanning was performed within a clinical timeframe using standard and prototype (UTE and DWI) sequences developed by the manufacturer for use on the clinical platform. It has previously been shown that this platform is suitable for preclinical work of this nature [21, 35], and can return functional MR parameters with good measurement repeatability across several imaging biomarkers. Repeated analysis by independent observers showed excellent repeatability of ROI positioning and all derived MR parameters except the pseudo-diffusion parameters from the IVIM diffusion model.

The results from the MT measurements were striking in their significance, with the presence of increased collagen leading to significant reductions in T_1_ measurements, as well as increased k_a_ and δ. After correcting for multiple comparisons, the correlations of these remained significant (p<0.0125). The MT ratio parameter, MTR, was correlated to picrosirius red stain fraction but fell short of significance. The similar parameter δ, less dependent on the influence of B_1_ [26], showed a stronger correlation and indicated that B_1_ effects should be accounted for when analysing MT data. The fibrous macromolecule collagen has a much shorter spin-lattice relaxation time T_1_ compared to normal tissue, and through magnetisation transfer to water protons reduces the apparent T_1_ of an imaging voxel dependent on the partial volume of collagen. The presence of the MT pulse saturates the collagen protons, and with transfer to the interacting water molecules, an additional and greater reduction occurs, giving much lower T_1s_. The apparent MT rate constant for the destruction of the water signal by the MT saturation, k_a_, is an empirical rather than a true rate constant [28], but does relate to the amount of collagen present, giving the observed correlation. Combining MT parameters using a PLSR analysis demonstrated a prediction error similar to that given from cross-validation using each parameter alone, indicating that different MT parameters provide statistically similar information on how collagen affects the tumour microenvironment. These results indicate that the MT measurement as performed was sensitive to the presence and proportion of collagen in the tumour, and can provide a non-invasive assessment of collagen content.

In diffusion-weighted imaging, the presence of collagen fibres will modify the diffusion characteristics of water molecules, providing additional barriers to free diffusion. In this study, the ADC and D values were negatively correlated with the picrosirius red staining, although with *p*-values short of significance (p=0.0274 and 0.0253, respectively), suggesting that the measurement of true diffusion is affected by the presence of fibrosis, in line with observations in hepatic fibrosis [36, 37]. These parameters were also found to contribute in the latent variables of the PLSR analysis, alongside MT parameters, although this model did not outperform the best individual MT parameters. The fibrous nature of collagen may also introduce heterogeneity to the diffusion hindrance, manifesting as a non-Gaussian diffusion component captured as a significant positive correlation of collagen presence with the pseudo-diffusion parameter fD*. The data for the pseudo-diffusion volume fraction f, often considered related to perfusion, showed no correlation with the endothelial marker CD31, which is likely reflective of the inherent difficulty in reliably fitting IVIM data, but also the complexity of tumour perfusion [30, 38]. In contrast, the non-significant correlation of f with necrosis (r=-0.607, p=0.0165) may suggest that f does not solely capture vascular fraction [23] and may be related to the degree of non-Gaussian diffusion introduced by the presence of collagen fibres [14]. The high CoV values associated with the pseudo-diffusion parameters, however, indicate that caution is required in interpreting these results.

In this study, the use of ultrashort echoes in order to visualise collagen did not give rise to a significant correlation. The conventional measurement of T_2_*_long_, using echo times longer than the relaxation time of collagen, showed a correlation to picrosirius red, suggesting that the overall voxel T_2_* is sensitive to the presence of fibrosis, and decreases with increasing collagen content.

The design of this study includes several limitations, which are nonetheless linked to its strengths. The use of clinical scanner hardware and imaging sequences means that while the scanner was not optimised for small animal studies, the techniques used were shown to be immediately translatable to clinical work. The mammary carcinoma model used in this work yielded tumours that varied considerably in presentation, growth rate, and composition; this reflects the clinical presentation of breast cancer and supports the potential of these results for clinical translation.

We have demonstrated the use of a multi-contrast MRI protocol to investigate the properties of chemically-induced mammary carcinoma in a preclinical setting, and have shown the potential of a clinical MT sequence to detect the presence of fibrosis non-invasively. Results from MT parameters outperformed those from multiple-b-value DWI and UTE imaging in detecting and quantifying intratumoral collagen, potentially providing information of biological relevance to support clinical assessment. Given that the presence of fibrosis is known to be a prognostic factor in mammary carcinoma, and may be induced following radiation therapy [39, 40], the results of this study support the inclusion of MT protocols in clinical breast MRI examinations.

## Electronic supplementary material

Supplementary Figure S1: Results of semi-automated segmentation and colour analysis of histological slices showing (left-to-right, for two tumours as per figures 1 and 2): picrosirius red stain, isolated picrosirius red stain, pimonidazole adduct stain, isolated pimonidazole adduct stain. The calculated stain maps are a binary mask, with false colour included only for display. (GIF 2832 kb)

High resolution image (TIFF 13964 kb)

Supplementary Figure S2: Loadings plots from PLSR analysis using (left panel) all MT parameters, and (right panel) all MR parameters for collagen stain prediction. In both cases, the regression favours a single latent variable (LV1) with loadings corresponding to observed correlations. The NRMSE for both models is comparable to individual MT parameters, although inclusion of ADC and D (individually non-signifcant) suggests complementary information may be available from DWI. (GIF 138 kb)

High resolution image (TIFF 79 kb)

## Abbreviations

ADC: Apparent diffusion coefficient
ARRIVE: Animal Research Reporting In Vivo Experiments
CoV: Coefficient of variation
DCE: Dynamic contrast-enhanced
DWI: Diffusion-weighted imaging
EPI: Echo-planar imaging
FID: Free induction decay
FITC: Fluorescein isothiocyanate
GRE: Gradient echo (also mGRE multiple gradient echo)
H&E: Haematoxylin and eosin
IVIM: Intravoxel incoherent motion
LOOCV: Leave-one-out cross-validation
LV: Latent variable
LR: Linear regression
MCMC: Markov chain Monte Carlo
MNU: *N*-methyl-*N*-nitrosourea
MRI: Magnetic resonance imaging
MT: Magnetisation transfer
MTR: Magnetisation transfer ratio
MVD: Microvessel density
NRMSE: Normalised root mean square error
PLSR: Partial least squares regression
ROI: Region of interest
TMJ: Temporomandibular joint
UTE: Ultrashort echo time
