# Supplementary figures and images for: Characterisation of fibrosis in chemically-induced rat mammary carcinomas using multi-modal endogenous contrast MRI on a 1.5T clinical platform
Source: Eur Radiol. 2017 Oct 16;28(4):1642–53. doi: 10.1007/s00330-017-5083-6 (PMC5834566; doi:10.1007/s00330-017-5083-6)

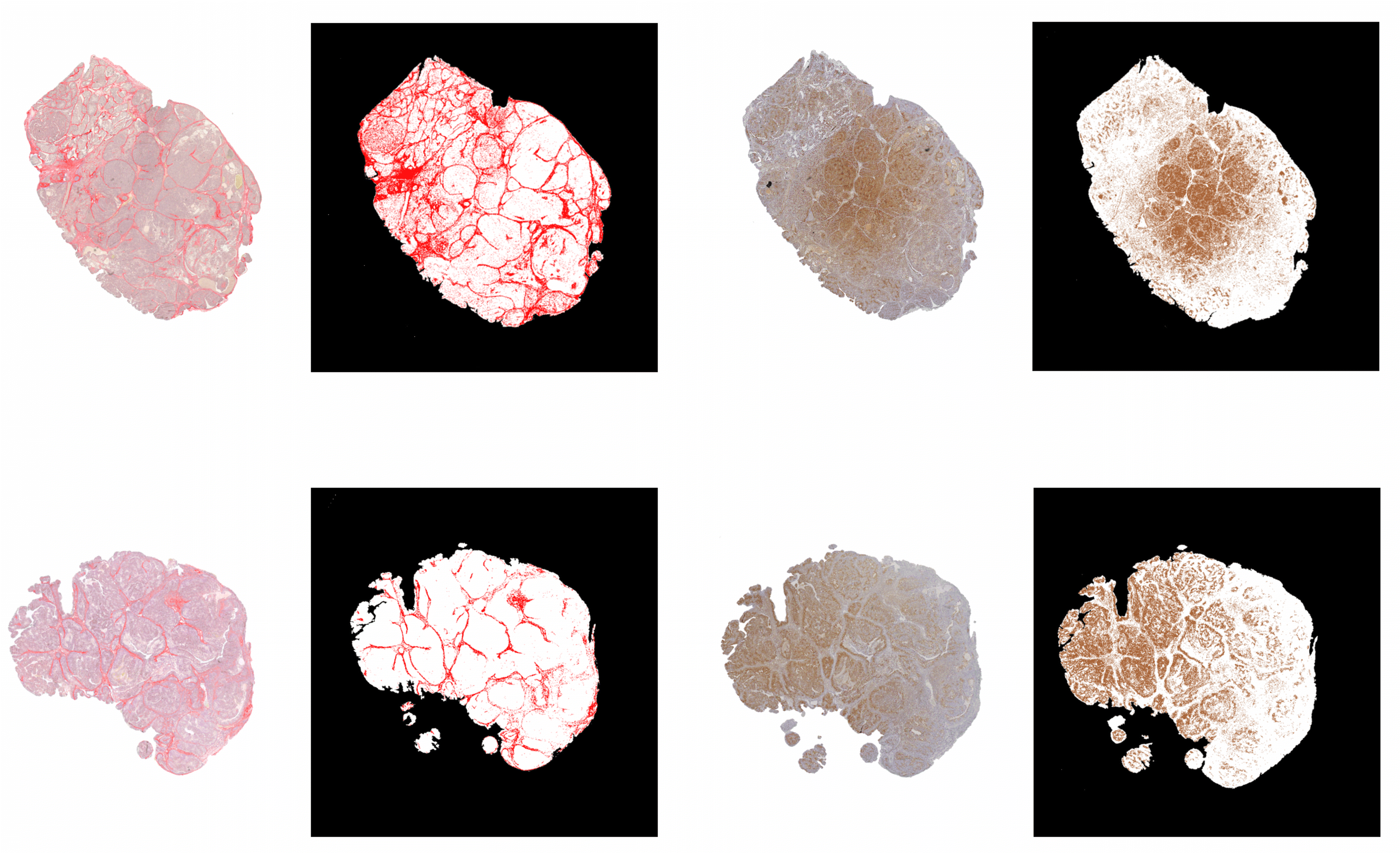

Supplement: Supplementary file 1 — Results of semi-automated segmentation and colour analysis of histological slices showing (left-to-right, for two tumours as per figures 1 and 2): picrosirius red stain, isolated picrosirius red stain, pimonidazole adduct stain, isolated pimonidazole adduct stain. The calculated stain maps are a binary mask, with false colour included only for display. (GIF 2832 kb) [file 330_2017_5083_Fig6_ESM.gif]

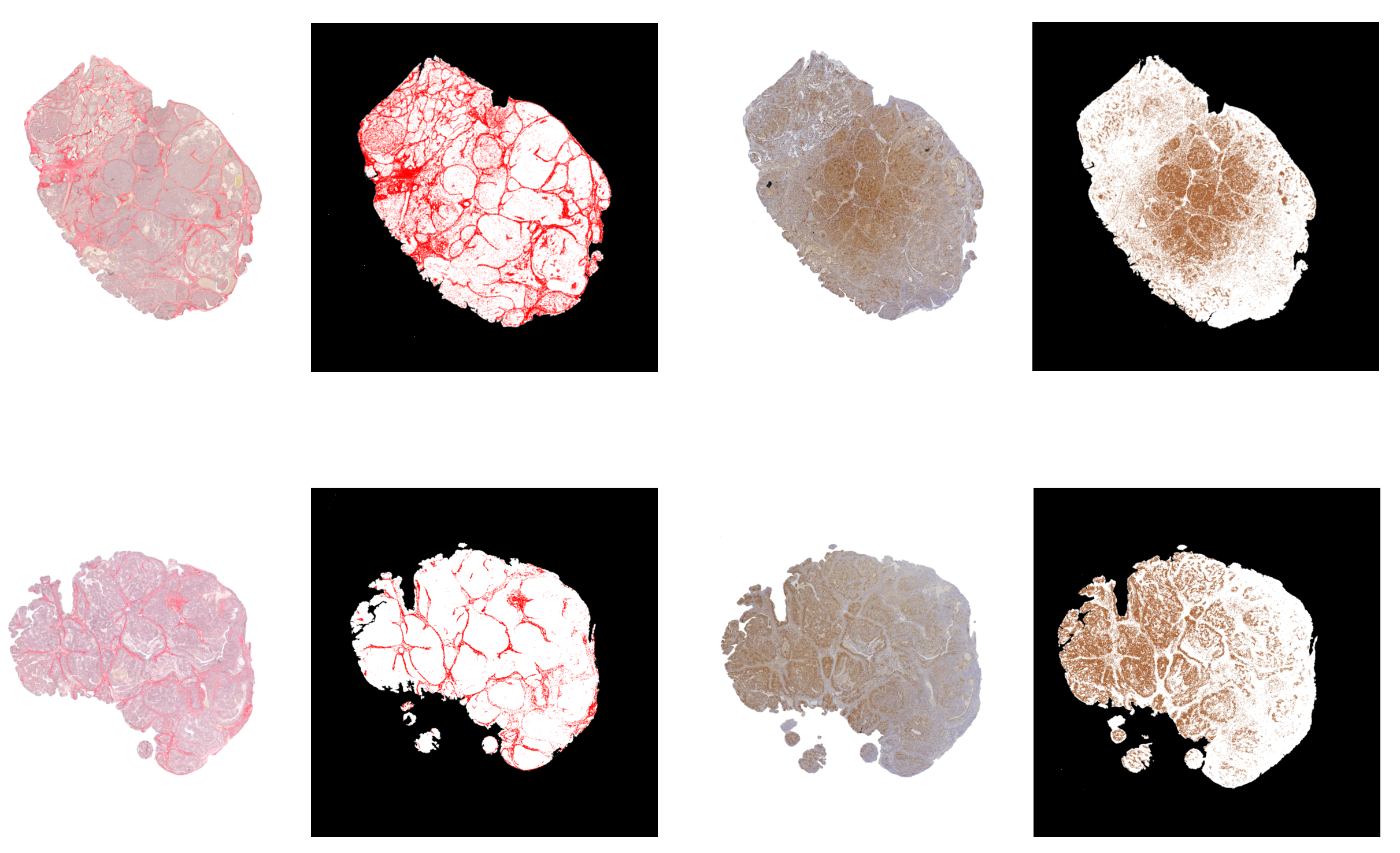

Supplement: Supplementary file 2 — High resolution image (TIFF 13964 kb) [file 330_2017_5083_MOESM1_ESM.tif]

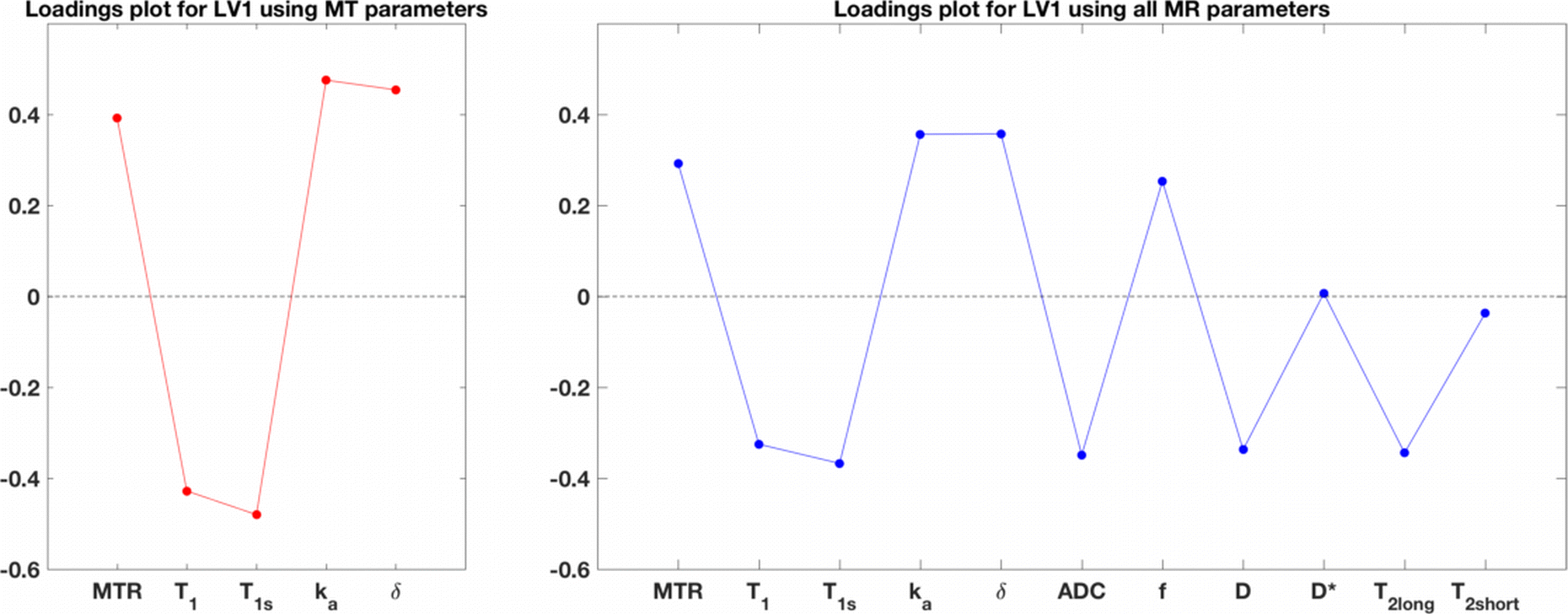

Supplement: Supplementary file 3 — Loadings plots from PLSR analysis using (left panel) all MT parameters, and (right panel) all MR parameters for collagen stain prediction. In both cases, the regression favours a single latent variable (LV1) with loadings corresponding to observed correlations. The NRMSE for both models is comparable to individual MT parameters, although inclusion of ADC and D (individually non-signifcant) suggests complementary information may be available from DWI. (GIF 138 kb) [file 330_2017_5083_Fig7_ESM.gif]

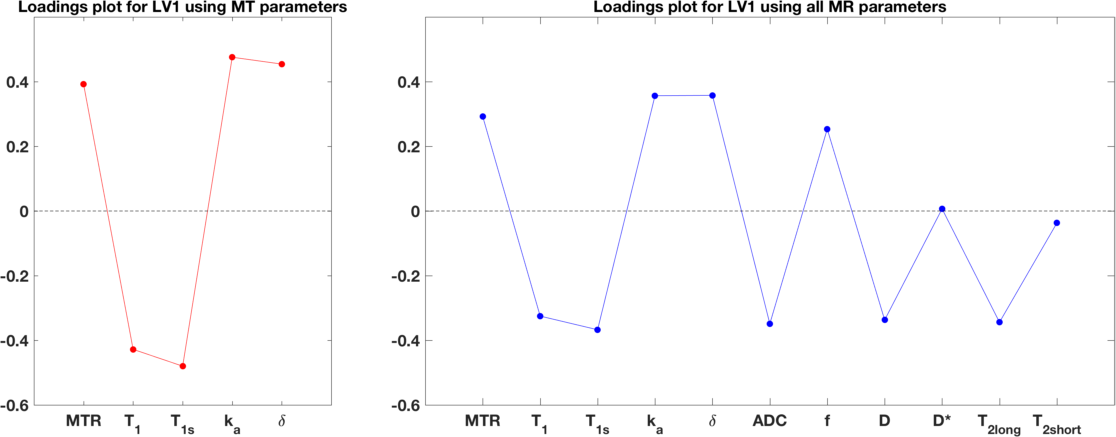

Supplement: Supplementary file 4 — High resolution image (TIFF 79 kb) [file 330_2017_5083_MOESM2_ESM.tif]
